# Resignation of Professor Meigs

**Published:** 1861-05

**Authors:** 


RESIGNATION OF PROFESSOR MEIGS.—The announcement of the retirement of this distinguished *savant* from the chair of obstetric medicine, which he had so ably filled for twenty years, in the Jefferson Medical College of this city, will be received with unfeigned regret by the many alumni and friends of that institution. His resignation was formally sent to the Board of Trustees in the latter part of February. He has thrown off the insignia of office at the close of a long, useful, and brilliant career, after having borne the heat and burden of the day for upward of half a century. Afraid lest advancing age should impair his capacity as a teacher, he was determined to quit the College in the fullness and vigor of his intellectual powers, ere the hand of decay had made the slightest impression upon him; his last course of lectures being marked by the same ability as the very best of the preceding. The valedictory which he delivered on his retirement from his chair will long be remembered by those who for the last time listened to his words as they gushed forth, warm and pathetic, from his lips, and sank deeply into the hearts of his fond and admiring pupils. He carries with him into private life the best wishes of his colleagues.

We understand that it is Dr. Meigs's intention to spend hereafter most of his time at his country residence, bearing the romantic Indian name of Hamanassett, about twenty miles southwest of Philadelphia, in the pursuits of literature and agriculture, and upon the composition of a work which he has long meditated upon the history of medicine; a task for which his extensive reading and acquaintance with ancient medical lore so well qualify him. We can conceive of no more delightful, dignified, or befitting occupation than this for one whose mind is so delicately organized as that of Dr. Meigs; and we trust that a kind Providence may render the evening of his life as happy and contented as its morning and meridian were honorable and prosperous.

The following minute was directed by the Faculty to be entered on their record on receiving the official announcement of the resignation of Dr. Meigs:—

The Faculty of the Jefferson Medical College of Philadelphia receive with deep sorrow the intelligence of the contemplated resignation of their distinguished colleague and friend, Professor Meigs.

For a long time he had expressed a desire to withdraw from the incessant labors of the most arduous branch of an arduous profession. Still, the Faculty had indulged the hope that it might be practicable for him to persist in his successful career of instructing others, which had ever been a labor of love with him, and to be content with abandoning, gradually or wholly, the practice of his profession.

He has decided otherwise, however, and sighs for retirement, away from the busy scenes to which he has been accustomed in civic life; and all must admit, that if any one can be entitled to a dignified leisure it is he.

The Faculty will part with him with intense and enduring regret. Never could any one have more closely applied himself to the execution of the responsible duties that have devolved upon him; and when sickness and sorrow have afflicted him, he was never absent from the amphitheatre at his accustomed hour, without feeling keenly the privation.

That he may reap every anticipated advantage from the course he has laid down for himself for the future, and may long be preserved to the cherished circle. to which his continued existence is so important, and to the world of science, is the fervent wish of his colleagues.

On commencement-day, March 9th, the graduating class presented to Dr. Meigs, through their chairman, Mr. Picot, an elegant portrait of himself, painted by Mr. Waugh, a distinguished Philadelphia artist. The ceremony was accompanied by an appropriate address by Mr. Picot, responded to in feeling terms by the retiring professor.

